# Comparative transcriptome profiling of a rice line carrying *Xa39* and its parents triggered by *Xanthomonas oryzae* pv. *oryzae* provides novel insights into the broad-spectrum hypersensitive response

**DOI:** 10.1186/s12864-015-1329-3

**Published:** 2015-02-21

**Authors:** Fan Zhang, Li-Yu Huang, Fan Zhang, Jauhar Ali, Casiana Vera Cruz, Da-Long Zhuo, Zheng-Lin Du, Zhi-kang Li, Yong-Li Zhou

**Affiliations:** Institute of Crop Sciences/National Key Facility for Crop Gene Resources and Genetic Improvement, Chinese Academy of Agricultural Sciences, 12 South Zhong-Guan-Cun Street, Beijing, 100081 China; Food Crops Research Institute, Yunnan Academy of Agricultural Sciences, Longtou Street, North suburbs, Kunming, Yunnan 650250 China; International Rice Research Institute, DAPO Box 7777, Metro Manila, The Philippines; Beijing Institute of Genomics, Chinese Academy of Sciences, No. 7 Beichen West Road, Chaoyang District, Beijing, 100029 China

**Keywords:** Rice, *Xanthomonas oryzae* pv. *oryzae*, *R* gene, Transcriptome sequencing

## Abstract

**Background:**

Bacterial blight, caused by *Xanthomonas oryzae* pv. *oryzae* (*Xoo*), is a devastating rice disease worldwide. *Xa39* is a resistance (*R*) gene with a broad-spectrum hypersensitive response (BSHR) to *Xoo*. Nevertheless, the molecular mechanisms of resistance mediated by *Xa39* remain unclear. In this study, the transcriptome profiling of a rice line carrying *Xa39* and its parents at the early stage of *Xoo* infection were investigated.

**Results:**

A rice introgression line H471 carrying *Xa39* exhibited a typical local hypersensitive response phenotype, accompanied by programmed cell death after inoculation with the *Xoo* Philippines’ race 9b. Transcriptome profiling of H471 and its parents at 1 and 2 days post-inoculation was performed using RNA sequencing. In total, 306 differentially expressed genes (DEGs) were identified in H471 compared with its recurrent parent Huang-Hua-Zhan after inoculation with *Xoo*. Among them, 121 (39.5%) genes, with functional enrichments that were related to defense response, protein amino acid phosphorylation, and apoptosis, were found to be constitutively expressed. The other 185 (60.5%) genes, with GO terms that belonged to defense response, were significantly responsive to *Xoo* infection in H471. Ten up-regulated and 12 down-regulated genes encoding intracellular immune receptors were identified in H471 compared with Huang-Hua-Zhan. LOC_Os11g37759, which was located in the fine-mapping region harboring *Xa39*, is a *Xa39* candidate gene. The putative BSHR-related co-regulatory networks were constructed using 33 DEGs from four functional groups, including gibberellic acid receptors and brassinosteroid regulators, which were differentially co-expressed with LOC_Os11g37759 in infected H471. Our results indicated that there might be cross-talk between the Xa39-mediated signal transduction cascades and the GA/BR signaling pathway, and that the defense mechanism was related to diverse kinases, transcription factors, post-translational regulation, and *R* genes.

**Conclusions:**

The present study provides the comprehensive transcriptome profile of a rice introgression line carrying *Xa39* and its parents, and identifies a set of DEGs involved in BSHR mediated by *Xa39*. These data provide novel insights into the regulatory networks of plant disease resistance mediated by *R* genes, and the identified DEGs will serve as candidates for *Xa39* cloning and for further understanding the molecular mechanism of BSHR.

**Electronic supplementary material:**

The online version of this article (doi:10.1186/s12864-015-1329-3) contains supplementary material, which is available to authorized users.

## Background

Bacterial blight (BB), caused by *Xanthomonas oryzae* pv. *oryzae* (*Xoo*), is a devastating rice disease worldwide [1]. The development and deployment of resistant cultivars carrying major resistance (*R*) genes has been the most effective approach for BB management [2]. However, rapid resistance loss in rice varieties carrying a single *R* gene is a problem for breeders [3-6]. Sustainable control measures for BB require a better understanding of resistance mechanisms in rice [7].

During the co-evolution between hosts and microorganisms, plants evolved a repertoire of *R* genes to defend themselves against pathogens by mounting effective, fine-tuned immune responses [8]. Like *Arabidopsis*, rice has evolved a two-layered innate immune system that includes pathogen-associated molecular pattern (PAMP)-triggered immunity (PTI) and effector-triggered immunity (ETI) [9]. PTI, the first layer of defense, is governed by pattern recognition receptors (PRRs) that recognize highly conserved PAMPs, triggering a relatively weak immune response that restricts colonization by invading organisms. To circumvent PTI, adapted pathogens can deliver effector molecules directly into the plant cell. Through co-evolution with pathogens, plants have accordingly developed intracellular immune receptors (R proteins) that can recognize the presence of certain pathogen effector molecules and trigger ETI [10]. In contrast to PTI, ETI, the second layer of defense, is a rapid and robust response, usually associated with localized programmed cell death (PCD), referred to as the hypersensitive response (HR), which is defined as a localized and rapid cell death response at sites of pathogen attack [11].

Functional genomic surveys of pathogen effectors have indicated that these proteins are highly diverse in sequence, as well as in molecular function [12-14]. However, the cognate R proteins in plants are structurally conserved. The numerous R proteins identified in *Arabidopsis thaliana* and in rice (*Oryza sativa*) have typically consisted of a variable amino terminus, a nucleotide-binding site (NBS) domain in the middle, and a leucine-rich repeat (LRR) domain at the carboxyl terminus. NBS-LRR type *R* genes in monocots, such as rice, usually carry a putative coiled-coil (CC-NBS-LRR) domain at the N-terminus [15].

Serving as a model system to elucidate the interactions between pathogens and monocotyledon plants, 39 *R* genes, 28 dominant and 11 recessive, conferring resistance to BB have been registered (http://www.shigen.nig.ac.jp/rice/oryzabase/gene/list) and identified [16], and six genes, *Xa1*, *xa5*, *xa13*, *Xa21*, *Xa26*/*Xa3*, and *Xa27*, have been cloned (http://www.shigen.nig.ac.jp/rice/oryzabase/gene/list). The first class of *R* genes contains receptor kinases, including the cloned *Xa21* and *Xa26* [17,18], but the molecules produced by *Xoo* that are recognized by XA21 and XA26 have not yet been characterized. *Xa1* represents the second major class of *R* genes, the NBS-LRR group. The gene for the elicitor signal from the pathogen has not been identified for *Xa1*. However, some genes encode diverse proteins that are expressed and trigger ETI after the recognition of the pathogen-delivered transcription activator-like (TAL) effector genes *avrXa27* [19-21]. Recently, there have been great advances in understanding the recognition between the R protein in rice and effector in *Xoo* including *Xa27* and AvrXa27 [22-24], *Xa10* and AvrXa10 (EBEAvrXa10) [21], and *Xa23* and TalC9b/AvrXa23 [20] however, research on how the R proteins trigger signal transduction cascades leading to HR in rice is still obscure.

*Xa39* is a novel dominant *R* gene with a broad-spectrum hypersensitive response (BSHR) to *Xoo* [16]. In this study, we compared transcriptome profiling by RNA sequencing (RNA-Seq) and identified differentially expressed genes (DEGs) in a rice introgression line (IL) H471 compared with its parents. Our results elucidated some interesting molecular mechanisms underlying BSHR mediated by R proteins in rice.

## Results

### Phenotype and DNA ladder detection of H471-*Xa39* in response to *Xoo*

**Figure 1:**
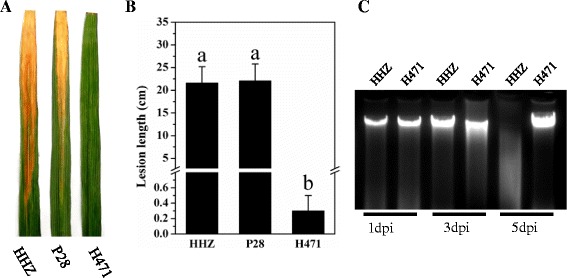
**Resistance reaction of the rice introgression line H471 carrying** ***Xa39*** **and parents Huang-** **Hua-** **Zhan** **(HHZ) and PSBRC28** **(P28) to** ***Xanthomonas oryzae*** **pv.** ***oryzae*** **(** ***Xoo*** **) PXO349.** **(A)** HHZ, P28, and H471 exhibited different lesion lengths when infected by PXO349 14 days post-inoculation (dpi). **(B)** Lesion lengths in HHZ, P28, and H471 plants at 14 dpi. The data represent the mean of nine independent plants in each line; vertical bars indicate standard deviation. Different letters above columns indicate statistically significant differences between means (*P* < 0.01, one-way analysis of variance, Dunnett’s multiple comparison test). **(C)** DNA fragmentation rate of HHZ and H471 at 1, 3, and 5 dpi.

Plants of H471, its recurrent parent Huang-Hua-Zhan (HHZ), and its donor parent PSBRC28 (P28) were inoculated with *Xoo* Phillipines’ race 9b (PXO349) at the tillering stage to evaluate their resistance reactions. H471 carrying *Xa39* displayed light brown along the wounded edges of clipped leaves at 3 days post-inoculation (dpi) and localized brown necrosis became more apparent at 5 dpi, exhibiting a typical HR. In contrast, a susceptible chlorotic symptom was visible on HHZ- and P28-infected leaves at 3 dpi, and water-soaked lesions rapidly spread along the clipped sites at 5 dpi. H471 was highly resistant to PXO349 with an average 0.3 ± 0.2 cm lesion length (LL) at 14 dpi. The two parents were highly susceptible to PXO349, with the LLs from 21.6 ± 3.6 to 22.1 ± 3.7 cm (Figures 1A and B).

DNA fragmentation rates of HHZ and H471 were detected after inoculation. DNA fragmentation was observed in both HHZ and H471 at 1 dpi (Figure 1C), and more fragmented DNA was observed in H471 than HHZ at 3 dpi. Notably, the DNA in HHZ was degraded at 5 dpi because of necrosis, whereas the DNA in H471 showed no changes, owing to the cessation of cell death.

### Gene expression profiling of three rice genotypes under inoculation and non-inoculation conditions

The total RNA from the inoculated leaves of H471, HHZ, and P28 at 1 and 2 dpi (designated as H471-1dpi, HHZ-1dpi, P28-1dpi, H471-2dpi, HHZ-2dpi, and P28-2dpi) and their corresponding non-inoculated controls (designated as H471-CK, HHZ-CK, and P28-CK) were paired-end sequenced using Illumina sequencing technology. A total of 10.5–25.0 million reads of 100 bp in length were generated for each sample, and the number of mapped reads was in the range of 8.6–20.2 million, with the matching ratio in the range of 81.1–82.6% (Table 1). The high-quality reads from individual libraries were mapped to the rice genome, and more than 21,785 mapped unique genes per library were determined simultaneously. The unique matching reads were used for further analysis. The high correlation (*R*^2^ = 0.87, *p* < 0.01) between RNA-seq and qRT-RCR expression values indicated that there was a high level of agreement between the approaches (Additional file 1: Figure S1).

**Table 1:** **Mapping results of RNA sequencing reads of the rice introgression line H471 carrying** ***Xa39*** **and parents Huang-Hua-Zhan** (**HHZ) and PSBRC28** (**P28) at 1 and 2 days post**-**inoculation** (**dpi) with** ***Xanthomonas oryzae*** **pv.** ***oryzae*** **and under non**-**inoculation conditions** (**CK)**

**Samples**	**Biological replication I**		**Biological replication II**		**Total mapped unique genes**
	**Total filtered pair-** **end reads**	**Total mapped reads** **(%)**	**Total filtered pair-** **end reads**	**Total mapped reads** **(%)**	
H471-CK	2 × 10,068,639	16,551,528 (82.2)	2 × 10,083,264	16,585,631 (82.3)	23625
HHZ-CK	2 × 8,894,670	14,403,823 (81.4)	2 × 9,326,860	15,241,546 (81.8)	23129
P28-CK	2 × 10,384,097	16,978,445 (81.9)	2 × 10,326,754	16,811,305 (81.4)	23693
H471-1dpi	2 × 5,232,832	8,601,699 (82.2)	2 × 11,822,492	19,355,542 (81.9)	22637
HHZ-1dpi	2 × 10,296,887	17,007,657 (82.6)	2 × 6,174,204	10,024,331 (81.2)	22725
P28-1dpi	2 × 12,505,956	20,248,936 (81.2)	2 × 11,484,590	18,600,880 (81.2)	23171
H471-2dpi	2 × 10,902,112	17,726,360 (81.3)	2 × 11,010,814	17,955,446 (81.6)	22954
HHZ-2dpi	2 × 5,873,150	9,522,108 (81.1)	2 × 5,453,981	8,865,653 (81.3)	21785
P28-2dpi	2 × 7,462,058	12,131,598 (81.3)	2 × 10,400,231	16,928,155 (81.4)	22556

The expressed genes in all samples were subjected to a cluster analysis. As shown in Additional file 2A: Figure S2, the three genotypes at 1 and 2 dpi, as well as the control, were separated from each other. The three control samples clustered into a group, and the inoculated samples clustered into another group, suggesting that most of the expressed genes had similar expression patterns in response to PXO349 infection, even in different genotypes. The inoculated H471 and HHZ clustered into a group, consistent with their similar genetic backgrounds, and samples at 1 and 2 dpi were in different subgroups, indicating that the gene expression responses to pathogen infection were dynamic.

To investigate the intrinsic differences in gene expression between the resistant and susceptible genotypes, the expression levels of genes in H471 were compared with those in HHZ and P28 under the non-inoculation condition. There were 1,161 DEGs identified between H471 and P28, and 343 DEGs between H471 and HHZ (Additional file 2B: Figure S2 and Additional file 3: Table S1). Between HHZ and P28, there were 1,286 DEGs because of the larger genetic difference between these two lines than between H471 and its parents. The genes with higher basal expression levels in H471 compared with HHZ were mainly functionally enriched in oxidoreductase activity, lyase activity, carboxylic acid metabolic process, response to stress, and cofactor binding (Additional file 4: Table S2).

### DEGs between the resistant and susceptible genotypes after inoculation with *Xoo*

**Figure 2:**
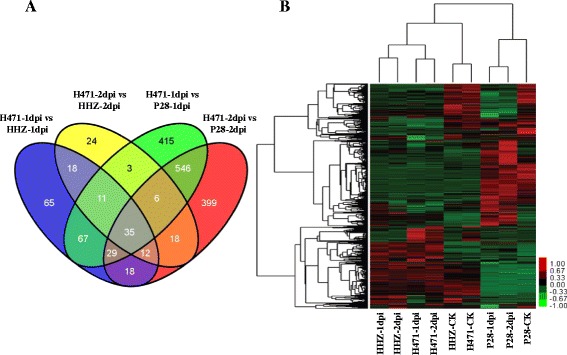
**Expression patterns of differentially expressed rice genes. (** **A)** Venn diagram showing the distribution of differentially expressed genes. **(B)** Hierarchical cluster of differentially expressed rice genes in nine samples. In the color panels, each horizontal line represents a single gene and the color of the line indicates the expression level of the gene relative to the mean center in a specific sample: high expression level in red, low expression level in green.

To determine the differences in the transcriptomes of the three genotypes responsive to *Xoo* infection, DEGs in the resistant genotype H471 were compared with its susceptible parents HHZ and P28 at 1 and 2 dpi. In total, 255 (113 up-regulated and 142 down-regulated) DEGs were found in H471-1dpi compared with HHZ-1dpi. Similarly, 1,112 (519 up-regulated and 593 down-regulated) DEGs in H471-1dpi compared with P28-1dpi were identified. Additionally, 127 (81 up-regulated and 46 down-regulated) and 1,063 (486 up-regulated and 577 down-regulated) DEGs were identified when H471-2dpi was compared with HHZ-2dpi and P28-2dpi, respectively (Additional file 3: Table S1). There were 142 genes in common between H471-1dpi and HHZ-1dpi, and H471-1dpi and P28-1dpi. Additionally, there were 71 in common between H471-2dpi and HHZ-2dpi, and H471-2dpi and P28-2dpi (Figure 2A). A hierarchical average linkage cluster analysis displayed the DEGs of the three genotypes in two genetic background-dependent clusters. The six samples of H471 and HHZ were in a cluster, and the three samples of P28 were in another cluster. The DEGs in H471-1dpi and H471-2dpi, HHZ-1dpi and HHZ-2dpi, H471-CK and HHZ-CK were in three different subgroups (Figure 2B), and the 306 DEGs in H471 compared with HHZ under inoculation conditions represent the transcriptomic profile resistant to BB mediated by *Xa39* (Figure 2A and Additional file 5: Table S3).

These 306 DEGs could be classified into two groups based on their expression patterns before and after the *Xoo* inoculation of H471. The first group contained 121 (39.5%) DEGs, which were differentially expressed under both non-inoculation and inoculation conditions. Among them, 64 genes were differentially up-regulated in H471 compared with HHZ after inoculation, whereas 57 genes were differentially down-regulated after inoculation (Additional file 5: Table S3). The first group is functionally enriched in defense response, protein amino acid phosphorylation, and apoptosis-associated proteins by GO analysis (Additional file 6: Table S4). The second group contained 185 DEGs that were responsive to *Xoo* infection at a minimum of one time point after inoculation. Among them, only one significant GO term (GO: 0006952, defense response), characterizing nine DEGs, was identified. These nine disease *R* genes included three up-regulated and six down-regulated genes responsive to *Xoo* infection (Additional file 5: Table S3).

### DEGs related to protein kinases in H471 and HHZ respond to *Xoo* infection

Protein kinases play central roles in signal recognition and the subsequent activation of plant defense mechanisms during pathogen infection. In this study, 31 genes encoding protein kinases were differentially expressed in H471 compared with HHZ, and 18 were responsive to *Xoo* infection (Additional file 7: Table S5). Among these DEGs, three types of receptor-like protein kinases (RLKs) were identified. The first group was composed of five genes encoding cysteine-rich receptor kinases (CRKs), which were differentially expressed in H471 compared with HHZ at 1 dpi (Additional file 7: Table S5). Among them, two genes encoding cysteine-rich RLK 10 (LOC_Os07g43560 and LOC_Os07g43570) were up-regulated, whereas the other three genes (LOC_Os04g30030, LOC_Os11g28104, and LOC_Os04g30040) were down-regulated. The second group was composed of two genes encoding the LRR protein kinases (LRKs) (LOC_Os08g14950 and LOC_Os11g36190), which were up-regulated in H471 compared with HHZ after inoculation with *Xoo*, and six genes encoding LRKs (LOC_Os03g56270, LOC_Os11g35500, LOC_Os08g14940, LOC_Os11g36140, LOC_Os11g36150, and LOC_Os03g28270), which were down-regulated in H471 compared with HHZ after *Xoo* inoculation (Additional file 7: Table S5). Of these, the expression of LOC_Os08g14950 was induced by *Xoo* infection, while the expression levels of the other four genes (LOC_Os08g14940, LOC_Os11g36140, LOC_Os11g36150, and LOC_Os03g28270) were inhibited in H471 at 1 dpi. The final group of RLKs consisted of seven genes encoding wall-associated kinases (WAKs), which were differentially expressed in H471 compared with HHZ at 1 dpi. Of the differentially expressed WAKs, *OsWAK38* (LOC_Os04g29680) was up-regulated in H471 compared with HHZ at 1 dpi, while the other six genes (LOC_Os04g30160, LOC_Os04g30110, LOC_Os04g29770, LOC_Os04g30250, LOC_Os04g29790, and LOC_Os04g29810) were down-regulated. These genes were all located in the introgressed fragments of chromosome 4 in H471 (Additional file 7: Table S5).

In addition to RLKs, two genes encoding calcium/calmodulin-dependent protein kinases (CPKs) were differentially expressed in H471 at 1 dpi. One gene (LOC_Os03g50330) was up-regulated, and the other (LOC_Os03g43440) was down-regulated (Additional file 7: Table S5).

### DEGs involved in phytohormone signaling pathways in response to *Xoo* infection

Plant hormones, such as salicylic acid (SA), jasmonate (JA), gibberellic acids (GAs), ethylene, and brassinosteroids (BRs), act as signals to trigger and mediate a diverse array of plant immune responses [19]. In this study, six genes related to SA, JA, or ethylene were up-regulated or down-regulated in H471 after inoculation with *Xoo*. However, none of them showed significantly different expression levels between H471 and HHZ after *Xoo* inoculation. (Table 2). However, a GA receptor, GID1L2 (LOC_Os07g44890), a BR signaling gene, *OsSERK1*/*OsBAK1* (LOC_Os08g07760), and a gene related to BR biosynthesis (LOC_Os03g40540), were up-regulated in H471 compared with HHZ after *Xoo* infection (Table 2 and Additional file 8: Figure S3).

**Table 2:** **Comparisons of the expression levels of representative hormone genes involved in rice innate immunity between the rice introgression line H471 carrying** ***Xa39*** **and the recurrent parent Huang**-**Hua**-**Zhan** (**HHZ)**

**Gene ID**	**Gene symbol**	**Note**	**H471-** **CK vs HHZ-** **CK**		**H471-** **1dpi vs HHZ-** **1dpi**		**H471**-**2dpi vs HHZ**-**2dpi**		**H471-** **1dpi vs H471-** **CK**		**H471-** **2dpi vs H471-** **CK**	
			**log2(** **FC)**	**Up/Down**	**log2** **(FC)**	**Up/Down**	**log2(** **FC)**	**Up/Down**	**log2** **(FC)**	**Up/Down**	**log2** **(FC)**	**Up/Down**
LOC_Os01g09800	*NH1*	SA pathway related gene	0.03	--	−0.30	--	−0.02	--	−0.90	down	−0.35	--
LOC_Os07g48820	*TGA2.1*/*OsbZIP63*	SA pathway related gene	0.12	--	0.12	--	0.01	--	0.01	--	0.16	--
LOC_Os05g49140	*OsMPK7*	JA responsive gene	−0.22	--	−0.02	--	0.11	--	1.48	up	1.65	up
LOC_Os08g39850	*LOX2*	JA biosynthesis	−0.24	--	−0.42	--	−0.14	--	−0.86	down	−0.64	--
LOC_Os01g60020	--	ABA/JA related gene	1.49	up	−0.21	--	0.29	--	−4.24	down	−3.72	down
LOC_Os02g43790	--	JA/Ethylene related gene	0.78	up	−0.28	--	0.13	--	−3.54	down	−3.37	down
LOC_Os05g48870	*ARF5*	Ethylene related gene	0.34	--	−0.13	--	0.09	--	1.28	up	0.70	--
LOC_Os07g44890	*GID1L2*	GA receptor	0.91	--	2.08	up	2.73	up	1.22	--	1.48	up
LOC_Os08g07760	*OsSERK1*	BR Signaling	0.34	--	1.28	up	0.99	up	0.68	--	0.53	--
LOC_Os03g40540	--	BR biosynthesis	0.19	--	1.18	up	0.60	--	1.83	up	0.99	up

### Differential expression of transcription factors (TFs) and genes possibly related to post-transcription regulation in H471 and HHZ

TFs are master regulators of gene expression and play key roles in the large-scale transcriptional reprogramming of plants in response to pathogen attacks. In this study, eight DEGs, five up-regulated and three down-regulated, encoding TFs in six families were identified in H471 compared with HHZ after infection by *Xoo*. Four of the genes were induced and one gene was inhibited by *Xoo* infection (Table 3). Five out of the eight TF genes, including LOC_Os03g42280 (B3 family), *OsIRO2* (LOC_Os01g72370, bHLH family), *OsMADS64* (LOC_Os04g31804, M-type family), *OsWRKY96* (LOC_Os07g40570, WRKY family), and LOC_Os07g39800 (HRT-like family), were up-regulated in H471. Two TFs belonging to the CO-like family (LOC_Os09g06464 and LOC_Os07g47140) and one TF belonging to the WRKY family (*OsWRKY4*, LOC_Os03g55164), were down-regulated in H471 (Table 3). Numerous studies in rice have indicated that WRKY TFs play complicated roles in plant defense signaling. For example, *OsWRKY53* and *OsWRKY22* overexpression lines are more resistant to *Magnaporthe grisea* [25]. Plants overexpressing *OsWRKY71* display enhanced resistance to virulent *Xoo* [26], and *OsWRKY62* is a negative regulator of both PTI and ETI [27]. Interestingly, in this study the expression levels of multiple WRKY TFs related to defense response were similar in infected H471 and HHZ (Additional file 9: Table S6). The up-regulated *OsWRKY96* in H471 compared with HHZ at 1 dpi was also not responsive to *Xoo* infection, while *OsWRKY4*, which was down-regulated in H471 compared with HHZ, was inhibited by *Xoo* infection (Table 3).

**Table 3:** **Differential expression of transcription process components in the rice introgression line H471 compared with the recurrent parent Huang**-**Hua**-**Zhan** (**HHZ) after inoculation with** ***Xanthomonas oryzae*** **pv.** ***oryzae***

**Gene ID**	**H471-** **1dpi vs HHZ-** **1dpi**		**H471-** **2dpi vs HHZ-** **2dpi**		**H471-** **1dpi vs H471-** **CK**		**H471-** **2dpi vs H471-** **CK**		**TF family/Annotation**
	**log2** **(FC)**	**Up/Down**	**log2** **(FC)**	**Up/Down**	**log2** **(FC)**	**Up/Down**	**log2** **(FC)**	**Up/Down**	
Transcription factors									
LOC_Os03g42280	3.6	up	3.3	up	1.1	up	0.7	--	B3
LOC_Os01g72370	0.6	--	1.5	up	−0.5	--	1.5	up	bHLH/OsIRO2
LOC_Os04g31804	2.4	up	1.8	up	1.5	up	1.4	up	M-type/OsMADS64
LOC_Os07g40570	1.2	up	0.7	--	−0.4	--	−0.7	--	WRKY/OsWRKY96
LOC_Os07g39800	1.5	up	0.9	--	−0.2	--	0.0	--	HRT-like/transcription repressor HOTR
LOC_Os09g06464	−1.0	down	−0.2	--	3.6	up	4.2	up	CO-like/OsCO3/CCT/B-box zinc finger protein
LOC_Os07g47140	−1.4	--	−1.8	down	−1.1	--	−1.7	--	CO-like/CCT/B-box zinc finger protein
LOC_Os03g55164	−1.0	down	−0.7	--	−1.2	down	−1.4	down	WRKY/OsWRKY4
Post-transcriptional processes									
LOC_Os03g53170	3.2	up	2.6	up	0.9	--	0.4	--	pentatricopeptide
LOC_Os08g12850	−1.1	down	−0.1	--	−0.9	--	−0.4	--	pentatricopeptide
LOC_Os02g43080	1.0	up	0.9	up	0.4	--	0.0	--	PPR repeat domain containing protein

Pentatricopeptide repeat (PPR) genes target effectors to the correct site on the correct transcripts, and are thus involved in many post-transcriptional processes [28]. In this study, three genes encoding PPRs were significantly expressed in infected H471 compared with HHZ (Table 3).

### Defense-related DEGs in the resistant and susceptible genotypes

**Figure 3:**
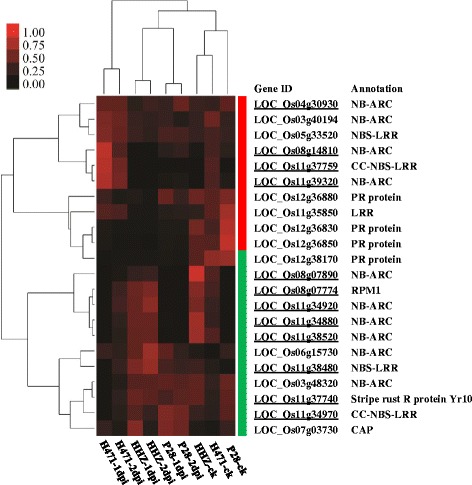
**Hierarchical cluster of 22 differentially expressed** ***R*** **genes in H471 compared with recurrent parent Huang-Hua-Zhan** **(HHZ) after** ***Xanthomonas oryzae*** **pv.** ***oryzae*** **(** ***Xoo*** **) PXO349 inoculation.** In the color panels, each horizontal line represents a single gene and the color of the line indicates the normalized expression level in a specific sample: high expression level in red, low expression level in black. The red and green bars on the right side of the heat map indicate the up-regulated and down-regulated genes, respectively, in H471 compared with HHZ after *Xoo* inoculation. The underlined gene ID indicates that H471 includes the allele from donor parent PSBRC28 (P28) at this locus.

The 10 up-regulated and 12 down-regulated *R* genes in H471 compared with HHZ after *Xoo* inoculation are shown in Figure 3 and Additional file 10: Table S7, respectively. Hierarchical clustering revealed that the two samples of H471 infected by *Xoo* clustered into one of two major subgroups, suggesting that H471 activates the expression of *R* genes more rapidly than its susceptible parents (Figure 3).

The 10 *R* genes up-regulated in H471 compared with HHZ included four genes belonging to the NB-ARC domain-containing protein family (LOC_Os03g40194, LOC_Os04g30930, LOC_Os08g14810 and LOC_Os11g39320), three genes encoding disease resistance proteins containing LRR/NBS-LRR domains (LOC_Os11g35850, LOC_Os05g33520 and LOC_Os11g37759), and three genes encoding pathogenesis-related proteins (PRs) (LOC_Os12g36880, LOC_Os12g36830 and LOC_Os12g36850) (Figure 3 and Additional file 10: Table S7). A further analysis indicated that four (LOC_Os04g30930, LOC_Os08g14810, LOC_Os11g39320, and LOC_Os11g37759) of the up-regulated *R* genes in H471 were co-localized in the introgressed regions (Figure 3 and Additional file 10: Table S7), implying roles in *Xoo* resistance.

PRs, which are involved in plant resistance to pathogens, are commonly induced in plants resistant to pathogens of viral, fungal, and bacterial origin [29]. For example, PR10/Bet v1, which is induced in hot pepper (*Capsicum annuum*) by incompatible interactions with *Xanthomonas campestris* pv. *vesicatoria*, may function as a ribonuclease. Subsequent phosphorylation of CaPR10/Bet v1 increases its ribonucleolytic activity to cleave invading viral RNAs, and this activity is important to its *in vivo* antiviral pathway [30]. Besides the three up-regulated genes encoding PRs, the expression of three genes (LOC_Os12g36830, LOC_Os12g36850, and LOC_Os12g36880) encoding the pathogenesis-related Bet v I family protein were inhibited in *Xoo*-infected H471.

Notably, two NBS-LRR class genes (LOC_Os05g33520 and LOC_Os11g37759) were induced in H471 after inoculation with *Xoo* (Additional file 8: Figure S3). LOC_Os05g33520 encoded a homolog resistant to phytophthora 1 in *Arabidopsis*, which positively regulates the *Phytophthora brassicae*-induced oxidative burst and enhances expression of defense-related genes [31]. LOC_Os11g37759, encoding a CC-NBS-LRR disease resistance protein, was expressed at a higher level in H471 compared with its parents before *Xoo* inoculation and was only induced by *Xoo* infection in H471 (Additional file 8: Figure S3). Notably, LOC_Os11g37759 was the only DEG out of 10 up-regulated *R* genes located in the 97.4-kb region (from 22233214 to 22330619 bp) on chromosome 11 harboring the *Xa39* gene based on our previous fine mapping [16], suggesting it was the most likely candidate gene of *Xa39*.

### Putative immunity mechanism of broad-spectrum resistance mediated by *Xa39*

**Figure 4:**
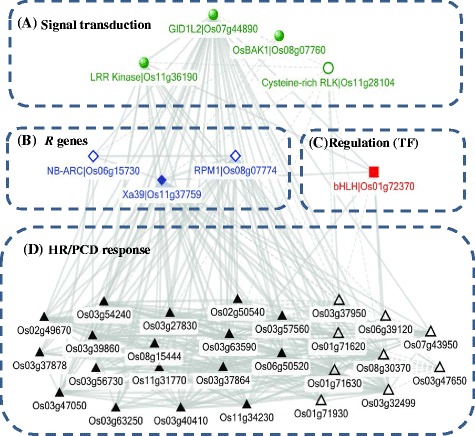
**Putative pathway or networks related to programmed cell death or the hypersensitive response in rice mediated by the** ***Xa39*** **candidate gene LOC_Os11g37759.** Out of all the DEGs in the rice introgression line H471 compared with recurrent parent Huang-Hua-Zhan (HHZ) after PXO349 inoculation, genes co-expressing with LOC_Os11g37759 (the *Xa39* candidate gene) were subjected to a co-expression analysis under biotic stress using the Pearson correlation coefficient (PCC) cutoff 0.70 in the Rice Oligonucleotide Array Database (ROAD) [32]. Co-expression networks were constructed based on the pairwise PCC of genes with a cutoff of 0.70 in ROAD. Four sub-groups, indicated with letters from A to D, were identified as their putative functions. The thickness of the edges is proportional to the pairwise PCCs, and the full and dotted edges indicate positive and negative PCCs, respectively, between the two genes in ROAD. Different shapes with different colors indicate different groups of genes based on the functional annotation. The full and open shapes indicate the genes were up-regulated and down-regulated, respectively, in H471 compared with in HHZ after PXO349 inoculation.

To explore the putative molecular networks related to the broad spectrum BSHR in H471, LOC_Os11g37759 (the *Xa39* candidate gene) was subjected to a co-expression analysis under biotic stress using the Pearson correlation coefficient (PCC) cutoff of 0.70 in the Rice Oligonucleotide Array Database (ROAD) [32]. In total, 33 genes overlapped with the 305 DEGs in H471 compared with HHZ after *Xoo* inoculation, and they were used to construct the putative genetic network related to PCD or HR that is mediated by *Xa39* (Figure 4 and Additional file 11: Table S8). In this network, the genes could be separated into four groups according to their putative functions. Group A was enriched in hormone signaling pathways and included LOC_Os08g07760 (encoding BR signaling-related OsBAK1), LOC_Os07g44890 (encoding gibberellin receptor GID1L2), and two genes, LOC_Os11g36190 and LOC_Os11g28104 (encoding receptor kinases). BAK1 is required to initiate PTI in *Arabidopsis* [33]. The *Xa39* candidate gene LOC_Os11g37759 (encoding a CC-NBS-LRR protein), and the other two co-expressed *R* genes were classified into group B. In rice, a broad-spectrum panicle blast-resistance gene encodes a CC-NBS-LRR protein [34]. The only gene, LOC_Os01g72370, in group C, which encoded a bHLH family TF, was involved in transcriptional regulation. Group D included two genes involved in ion transport (Additional file 8: Figure S3) and 17 genes encoding proteins of unknown functions.

## Discussion

The BSHR phenotype of rice IL carrying *Xa39* is different from that of its parents. It is possible that a rare unequal rearrangement event, insertion, or deletion has occurred, which causes the novel resistance [16]. In our previous study, two genes encoding CC-NBS-LRR disease resistance proteins, LOC_Os11g37740 and LOC_Os11g37759, were mapped in the 97.4-kb region harboring the *Xa39* gene on rice chromosome 11 [16]. In this study, LOC_Os11g37759 was differentially up-regulated in H471 compared with its parents, suggesting that the hypothetical mechanism of *Xa39* might involve this gene.

Among 39 *R* genes resistant to BB, *Xa39* and *Xa23* exhibit similar BSHR, with LLs shorter than 1 cm [16,35]. *Xa21*, which is discussed as a putative PRR, and the signaling network mediated by Xa21 have been studied extensively [19]. *Xa21*-containing rice lines also have broader resistance spectra, but the LLs were longer than 5 mm when inoculated with *Xoo* strains possessing strong virulence, including PXO349. This is in accordance with PTI playing a pivotal role in the defense against a broad spectrum of potential pathogens, but triggering a relatively weak immune response in contrast to ETI [9,19]. To date, it has been reported that *Xa23* can be recognized by the TAL effector talC9b/avrXa23 and triggers resistance to *Xoo* [20]; however, the signaling network leading to HR still remains to be elucidated. In this study, the comparative transcriptome profiling of H471 and its parents provided several interesting insights into the molecular mechanisms of BSHR in rice.

First, the resistance mediated by *Xa39* might not be related to ion fluxes and oxidative bursts. The signal-specific activation of plant PRRs by PAMPs leads to a series of cellular processes, including ion fluxes, oxidative bursts by the production of reactive oxygen species (ROS), activation of downstream MAPK cascades, transcriptional changes, and the production of antimicrobial compounds, such as PR and phytoalexins [36]. In this study, six genes encoding PRs were differentially expressed; however, there were no significant differences in the expression levels of the representative PRRs of PTI, genes related to oxidative burst, or downstream genes related to cell death due to ion fluxes and ROS in H471 compared with HHZ after inoculation with *Xoo* (Additional file 8: Figure S3 and Additional file 12: Table S9). This suggests that hypersensitive cell death at sites of *Xoo* infection did not result from the signal-specific activation of PRRs in H471.

Second, GA and BR signaling pathways may be involved in BSHR mediated by *Xa39*. It is generally accepted that SA plays a major role in activating defenses against biotrophic pathogens, whereas JA is usually associated with defenses against necrotrophic pathogen attacks [37]. Grewal et al. [38] reported that JA and ethylene signaling were involved in the early defense of rice cells, and four overlapping genes related to JA or ethylene were up-regulated or down-regulated in H471 after inoculation with *Xoo* (Table 2). However, none of them showed a significantly different expression level between H471 and HHZ after *Xoo* inoculation (Table 2). Interestingly, several genes related to GA and BR signaling were differentially expressed in H471 compared with its parents. In rice, it was inferred that the GA regulator OsSERK2 positively regulates immunity mediated by XA21 and XA3 [19,39]. LOC_Os11g37759, encoding a CC-NBS-LRR protein, is structurally related to XA21 and XA3, and OsSERK1/OsBAK1 is the closest paralog to OsSERK2 in rice. Interestingly, we found that there was a strong co-expression of LOC_Os11g37759 and OsSERK1/OsBAK1 (Figure 4). In rice, SLR1 (DELLA family), a key regulator of GA signaling, functions in disease resistance to *Xoo* as a positive regulator through cross-talk with the JA signaling pathway via the GA receptor GID1 [19]. In this study, there were no significant differences in the expression levels of SLR1 and GID1 between H471 and HHZ; however, the GA receptor GID1L2 was differentially co-expressed with LOC_Os11g37759. The BR signaling-related gene *OsBAK1* (LOC_Os08g07760) and BR biosynthesis-related gene LOC_Os03g40540 were expressed at higher levels in H471 compared with HHZ after *Xoo* infection (Table 2). The activation of BR signaling inhibits PTI by the BAK1-independent recognition of the fungal PAMP chitin [40]. Although the type of immunity activated by *Xa39* remains a mystery, the differential expression levels of these hormone regulators suggests that there might be cross-talk between the *Xa39*-mediated signal transduction cascades and the GA/BR signaling pathway.

Finally, diverse categories of DEGs related to signal transduction and regulation were identified. WAKs span the plasma membrane, allowing cells to recognize and respond to their extracellular environment [41]. CPKs can regulate downstream components of complex immune and stress signaling networks as positive or negative regulators [42]. For example, *OsCPK12* is involved in both salt-stress tolerance and blast disease resistance in rice [43]. Several CRKs are reportedly associated with resistance to pathogen infection and activation of PCD in *Arabidopsis* [44-46], barley [47], and wheat [48]. In this study, DEGs encoding LRKs, WAKs, CPKs, and CRKs were identified at 1 and 2 dpi, indicating that a series of signaling events was regulated by complex communications at the early stage of infection in H471. TFs in several families such as WRKY, MADs box, and NAC are involved in biotic and abiotic stresses, as well as pathogen invasion [23]. In this study, besides the two TFs in the WRKY family, TFs in B3, bHLH/OsIRO2, M-type, HRT-like, and CO-like families were differentially expressed in H471 compared with their parents, suggesting that functions of diverse TFs in plant resistance remain to be elucidated.

HR accompanying PCD at the site of attack is an effective defense strategy against pathogens and nematodes that feed on live plant cells [36]. However, unlike the nucleotide binding oligomerization domain (NOD)-like receptors (NLRs) and LRR-containing proteins in animals, which are known to trigger cell apoptosis through the activity of caspases that activate proinflammatory cytokines [49], plants lack the homologous caspases. The hypothesis that R-protein-mediated PCD might involve the perturbation of multiple cellular processes came from a report showing that in *A. thaliana*, the resistance conferred by the R protein RPP4 against the obligate biotroph *Hyaloperonospora arabidopsidis* is not mediated by a single gene, but rather by multiple downstream genes that encode proteins (mostly enzymes with very diverse functions) [50]. The DEGs identified in this study, which might be involved in perception, signal transduction, transcriptional regulation, and defense-related genes, will be studied to determine their functions and to understand the molecular networks involved in the BSHR mediated by *R* genes in rice.

## Conclusions

The BB *R* gene *Xa39*, which is able to trigger a resistance response to *Xoo* characterized by PCD in rice plants, activates DEGs encoding proteins with diverse functions leading to BRHR. These results provide novel insights into the regulatory networks of plant resistance mediated by R proteins.

## Methods

### Plant materials and artificial inoculation

Three rice genotypes were used in this study. HHZ, with a high yield and good quality, is a widely used *indica* inbred rice line in southern China. P28 is an *indica* rice variety from the Philippines. H471, carrying *Xa39*, is a BC_1_F_6_ IL with a few chromosomal fragments introgressed from the donor parent P28 into the background of the recurrent parent HHZ. A genome-wide single nucleotide polymorphism analysis by re-sequencing showed that H471 differs from HHZ at 26 genomic segments, which originate from P28, with sizes in the range of 57–6,057 kb (Additional file 13: Figure S4). The recombination events were judged as described in Huang et al. [51], using the genome-resequencing data of HHZ, P28, and H471 (unpublished data). Seeds of H471, HHZ, and P28 were sown in a seedling nursery and 30-day-old seedlings were transplanted into a screenhouse of the Institute of Crop Sciences, Chinese Academy of Agricultural Sciences, Beijing, China. There were nine plants in each row, with a spacing of 20 × 17 cm. The Philippine’s representative strain of *Xoo*, PXO349, was used to artificially inoculate H471 and its parents. The strain was incubated on peptone sucrose agar at 30°C for 2 days, and the inoculum was prepared by suspending the bacterial mass in sterile water at a concentration of 10^8^ cells mL^−1^. Five plants of each line were inoculated with PXO349 in the four to five uppermost leaves of each plant using the leaf-clipping method [52] in three replications at the tillering stage (plant age of 65 days). LLs were measured on all inoculated leaves at 14 dpi when the lesions were stable.

### DNA fragmentation of HHZ and H471 leaves infected by PXO349

Leaves of HHZ and H471 inoculated by PXO349 at 1, 3, and 5 dpi were collected for a DNA fragmentation analysis. A 5-mm leaf fragment under the incision was collected and extracted using the DNeasy Plant Mini Kit (Cat. No.69106) following the manufacturer’s manual.

### Preparation of samples for RNA-Seq

Leaves of plants were clipped with sterile water and PXO349, respectively. 1-cm-long leaf tips from H471, HHZ, and P28 were dissected at 0 day after treatment with water, and at 1 day and 2 days after inoculation with PXO349. Two replicate leaf samples were taken for each rice line treatment at each time point. Samples of H471, HHZ, and P28 collected at 0 dpi were used as control and named as H471-CK, HHZ-CK, and P28-CK, respectively. Samples of H471, HHZ, and P28 collected at 1 dpi were named as H471-1dpi, HHZ-1dpi, and P28-1dpi, respectively; and samples collected at 2 dpi were named as H471-2dpi, HHZ-2dpi, and P28-2dpi, respectively. All samples were immediately frozen in liquid nitrogen after collection, and stored at −80°C.

### RNA extraction, mRNA-Seq library construction, and sequencing

For all RNA-Seq samples, total RNA was extracted with TRIzol Reagent (Invitrogen, Carlsbad, CA, USA), and quantified using a Qubit RNA assay kit (Applied Biosystems, Foster City, CA, USA). RNA integrity was checked using an Agilent 2100 Bioanalyzer (Agilent Technologies, Santa Clara, CA, USA). Total mRNAs were isolated by oligo(dT) selection using Dynal® magnetic beads (Invitrogen). The paired-end fragment library was constructed following the TruSeq RNA Sample Preparation kit (Illumina, San Diego, CA, USA) protocol with minor modifications. Cluster generation of the produced libraries was performed using cBot, and sequenced on a HiSeq 2000 platform (Illumina) with paired-end 100-bp reads. Primary data analysis and base calling were performed using the Illumina instrument software.

### Transcriptome data processing and analysis

All primary sequencing data are available at the GEO database under the accession number GSE62488 (http://www.ncbi.nlm.nih.gov/geo/query/acc.cgi?acc=GSE62488). Low-quality nucleotides (< Q20) were trimmed from raw sequences for each sample, and then pair-end reads with lengths less than 30 bp were removed using an in-house Perl script. Retained high-quality pair-end reads of rice for each sample were mapped to the rice genome of the Rice Genome Annotation Project (RGAP) at MSU [53] using TopHat [54], and assembled with Cufflinks [55] to construct unique transcript sequences using the parameter –g –b –u –o. Cuffcompare was used to compare the assembled transfrags of each sample to the reference annotation, and to build a non-redundant transcript dataset among the samples [55]. The number of mapped clean reads for each gene was counted and normalized into the reads per kilobase per million value [56]. Next, Cuffdiff was used to identify DEGs, and genes with *P* values ≤ 0.001 were marked as significantly different between the two samples [55]. GO terms of rice DEGs were identified in accordance to the methodology described by Du et al. [57]. A Venn diagram was built using software available online (http://bioinfogp.cnb.csic.es/tools/venny/index.html) [58].

### Confirmation of the DEGs by quantitative real-time RT-PCR (qRT-PCR)

To validate the Illumina sequencing results, a subset of DEGs were verified by qRT-PCR (n = 108). An independent set of samples was collected at 6, 12, 24, 36, 48, and 60 h after inoculation with PXO349 to use for the expression analysis of some important DEGs. The sequence for each rice gene was obtained from the MSU rice database [53]. Sequences from each gene were used to design primers using Primer 5 software (http://frodo.wi.mit.edu/; Additional file 14: Table S10). Independent biological repetitions of each experiment were performed in triplicate. Expression levels of 17 rice genes were tested in 20-μL reactions using the SYBR® Green PCR Master Mix kit (Applied Biosystems) following the manufacturer’s protocol via an ABI Prism 7900 Sequence Detection System (Applied Biosystems).

### Availability of supporting data

The sequencing raw data of this article are available through a GEO database at the NCBI (http://www.ncbi.nlm.nih.gov/geo/query/acc.cgi?acc=GSE62488). And the data sets supporting the results of this article are included within the article and its additional files.

## Additional files

Additional file 1: Figure S1. Comparison of transcription levels measured by RNA-seq and quantitative real-time reverse transcription-PCR (qRT-PCR) assays. Containing a scatter plot comparing transcription levels as measured by RNA-seq and quantitative real-time reverse transcription-PCR (qRT-PCR) assays. The gene expression values were transformed to the log2 scale. The qRT-RCR log2-values (X-axis) were plotted against the FPKM log2-values (Y-axis). Additional file 2: Figure S2. Expression patterns of rice genes in nine libraries. Containing hierarchical cluster analysis of nine sample pools of the expressed genes and a Venn diagram. **(A)** Clustering of expressed rice genes. **(B)** Venn diagram showing the distribution of differentially expressed genes among the rice introgression line H471 vs. the recurrent parent Huang-Hua-Zhan (HHZ), H471 vs. the donor parent PSBRC28 (P28), and HHZ vs. P28 under control conditions. Additional file 3: Table S1. Summary of differentially expressed genes in the rice introgression line H471, the recurrent parent Huang-Hua-Zhan (HHZ) and the donor parent PSBRC28 (P28). Listing the summary of differentially expressed genes in H471, HHZ and P28. Additional file 4: Table S2. GO enrichment analysis of genes with higher basal expression levels in the rice introgression line H471 compared with the recurrent parent Huang-Hua-Zhan (HHZ). Containing a list of GO enrichments of genes with higher basal expression levels in H471 compared with HHZ. Additional file 5: Table S3. Differentially expressed genes in the rice introgression line H471 compared with recurrent parent Huang-Hua-Zhan (HHZ) after inoculation with *Xanthomonas oryzae* pv. *oryzae* (*Xoo*) PXO349. Containing a list of differentially expressed genes in H471 compared with HHZ under post-inoculation conditions. Additional file 6: Table S4. GO enrichment analysis of the differentially expressed genes unresponsive to *Xanthomonas oryzae* pv. *oryzae* (*Xoo*) PXO349 infection in the rice introgression line H471 compared with the recurrent parent Huang-Hua-Zhan (HHZ) under post-inoculation condition. Containing GO enrichment analysis result of the differentially expressed genes unresponsive to PXO349 infection in H471 compared with in HHZ under post-inoculation conditions. Additional file 7: Table S5. Differential expression of components in signal transduction pathways in the rice introgression line H471 compared with the recurrent parent Huang-Hua-Zhan (HHZ) after *Xanthomonas oryzae* pv. *oryzae* (*Xoo*) PXO349 inoculation. Containing differentially expressed genes involved in signal transduction pathways in H471 compared with HHZ after PXO349 inoculation. Additional file 8: Figure S3. Expression level of 17 genes involved in five classes by quantitative real-time reverse transcription-PCR (qRT-PCR) at control condition and six time points after *Xanthomonas oryzae* pv. *oryzae* (*Xoo*) PXO349 infection. Showing the expression level of 17 genes involved in five classes by qRT-PCR under control conditions and six time points after *Xoo* infection. **(A)** Brassinosteroid signaling-related genes. **(B)** Genes involved in hypersensitive cell death due to ion fluxes or reactive oxygen species bursts. **(C)** *R* genes. **(D)** Genes involved in ion transport. **(E)** Genes encoding pathogenesis-related proteins. Error bars indicate standard deviation. Actin was used as an endogenous control. Additional file 9: Table S6. Ten genes of the WRKY family expressed at the similar levels in the rice introgression line H471 and the recurrent parent Huang-Hua-Zhan (HHZ) after *Xanthomonas oryzae* pv. *oryzae* (*Xoo*) PXO349 infection. Containing 10 genes of the WRKY family expressed at the similar levels in H471 and HHZ after PXO349 infection. Additional file 10: Table S7. Differentially expressed defense-related genes in the rice introgression line H471 compared with the recurrent parent Huang-Hua-Zhan (HHZ) infected by *Xanthomonas oryzae* pv. *oryzae* PXO349. Containing differentially expressed defense-related genes in H471 compared with HHZ infected by *Xanthomonas oryzae* pv. *oryzae*. Additional file 11: Table S8. Gene list of putative networks related to programmed cell death or the hypersensitive response mediated by *Xa39* candidate gene LOC_Os11g37759. Containing genes of putative networks related to programmed cell death or the hypersensitive response mediated by the *Xa39* candidate gene LOC_Os11g37759 based on a co-expression analysis. Additional file 12: Table S9. Comparison of expression levels of representative rice genes related to rice innate immunity between the rice introgression line H471 and the recurrent parent Huang-Hua-Zhan (HHZ). Containing comparisons of expression levels of representative rice genes related to rice innate immunity between H471 and HHZ. Additional file 13: Figure S4. Recombination map of the rice introgression line H471. Containing introgressed bins of H471 based on a genome-wide single nucleotide polymorphism analysis by re-sequencing. Additional file 14: Table S10. Information on primers used in the qRT-PCR analysis. Containing information on primers used in the qRT-PCR analysis.
