# Supplementary material for: Comparative transcriptome profiling of a rice line carrying Xa39 and its parents triggered by Xanthomonas oryzae pv. oryzae provides novel insights into the broad-spectrum hypersensitive response
Source: BMC Genomics. 2015 Feb 21;16(1):111. doi: 10.1186/s12864-015-1329-3 (PMC4349310; doi:10.1186/s12864-015-1329-3)
Supplement: Additional file 1: Figure S1. — Comparison of transcription levels measured by RNA-seq and quantitative real-time reverse transcription-PCR (qRT-PCR) assays. Containing a scatter plot comparing transcription levels as measured by RNA-seq and quantitative real-time reverse transcription-PCR (qRT-PCR) assays. The gene expression values were transformed to the log2 scale. The qRT-RCR log2-values (X-axis) were plotted against the FPKM log2-values (Y-axis). [file 12864_2015_1329_MOESM1_ESM.pdf]

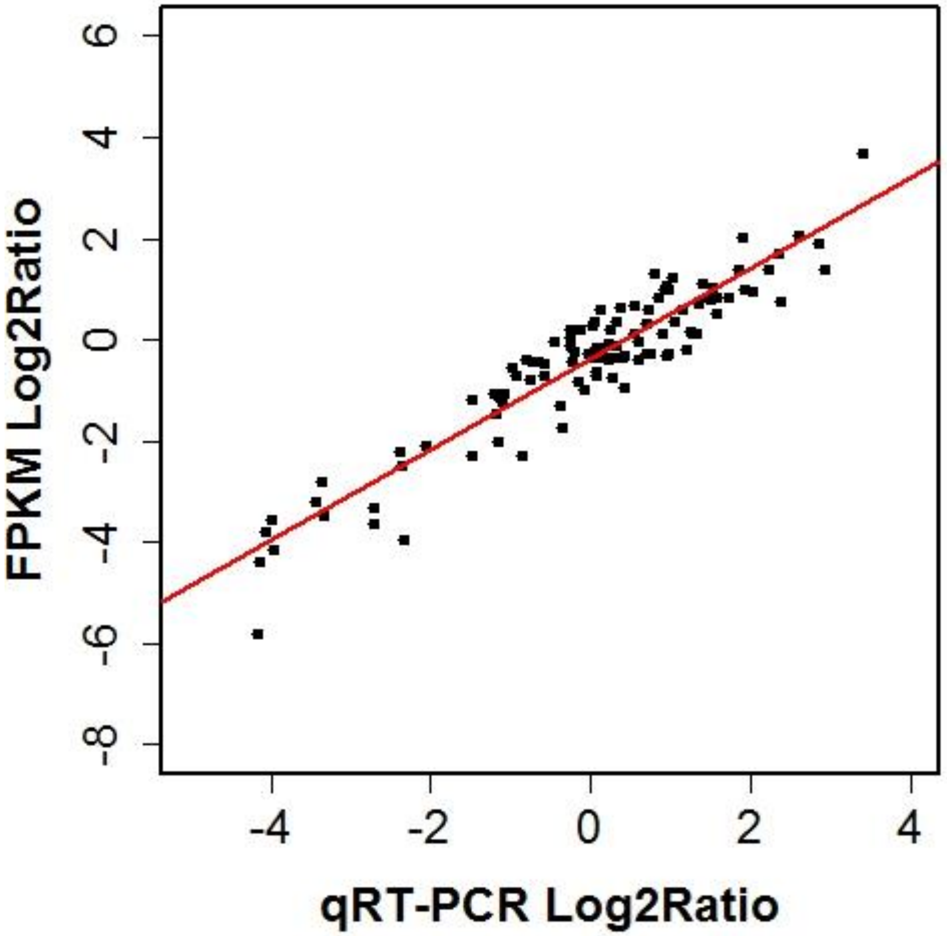

Additional file 1. Comparison of transcription levels measured by RNA-seq and quantitative real-time reverse transcription-PCR (qRT-PCR) assays. The gene expression values were transformed to the log2 scale. The qRT-RCR log2-values (X-axis) were plotted against the FPKM log2-values (Y-axis).
