# Supplementary material for: Comparative transcriptome profiling of a rice line carrying Xa39 and its parents triggered by Xanthomonas oryzae pv. oryzae provides novel insights into the broad-spectrum hypersensitive response
Source: BMC Genomics. 2015 Feb 21;16(1):111. doi: 10.1186/s12864-015-1329-3 (PMC4349310; doi:10.1186/s12864-015-1329-3)
Supplement: Additional file 2: Figure S2. — Expression patterns of rice genes in nine libraries. Containing hierarchical cluster analysis of nine sample pools of the expressed genes and a Venn diagram. (A) Clustering of expressed rice genes. (B) Venn diagram showing the distribution of differentially expressed genes among the rice introgression line H471 vs. the recurrent parent Huang-Hua-Zhan (HHZ), H471 vs. the donor parent PSBRC28 (P28), and HHZ vs. P28 under control conditions. [file 12864_2015_1329_MOESM2_ESM.pdf]

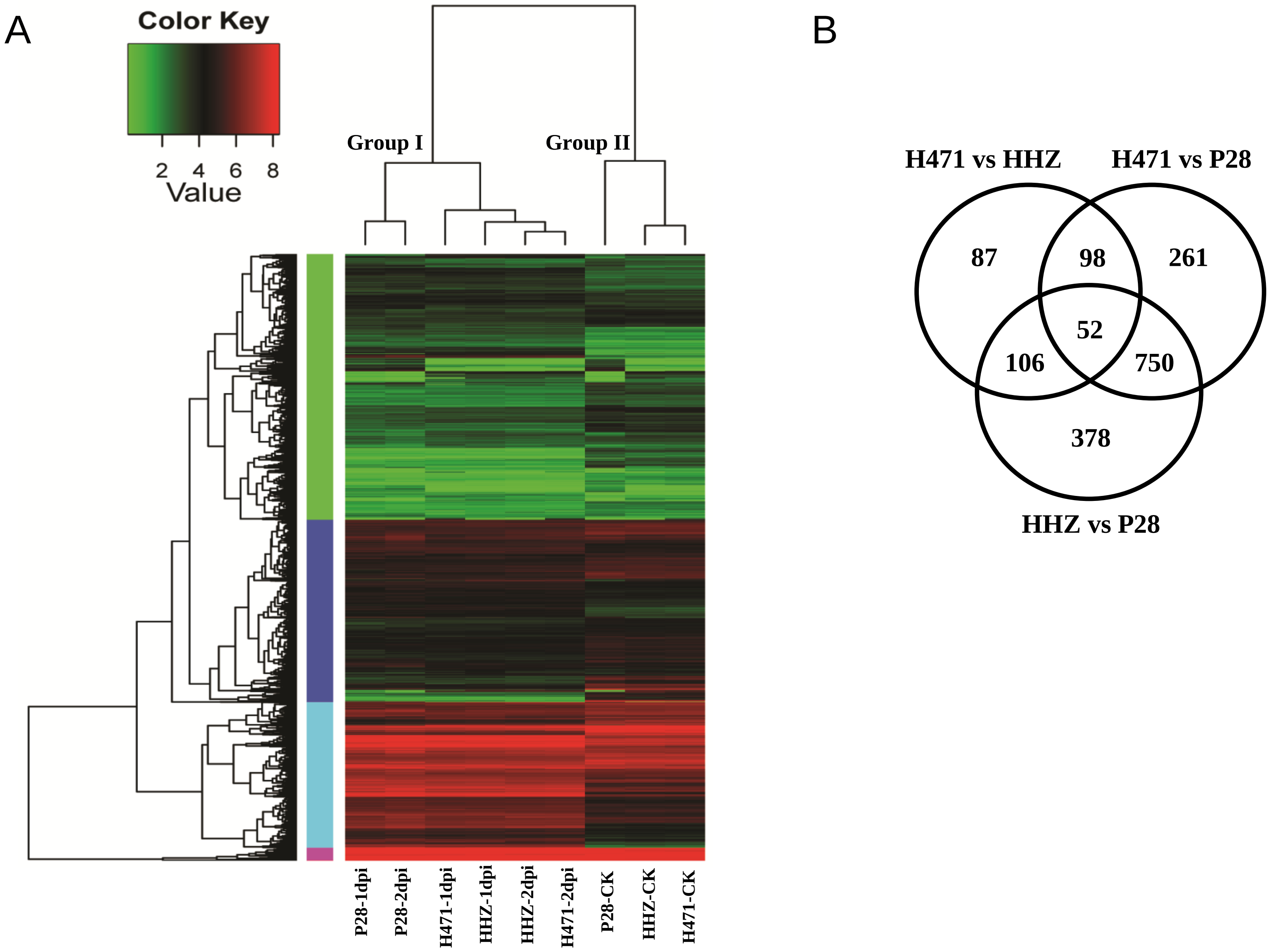

Additional file 2. Expression patterns of rice genes in nine libraries. (A) Clustering of expressed rice genes. (B) Venn diagram showing the distribution of differentially expressed genes among the rice introgression line H471 vs. the recurrent parent Huang-Hua-Zhan (HHZ), H471 vs. the donor parent PSBRC28 (P28), and HHZ vs. P28 under control conditions.
