# Supplementary material for: Comparative transcriptome profiling of a rice line carrying Xa39 and its parents triggered by Xanthomonas oryzae pv. oryzae provides novel insights into the broad-spectrum hypersensitive response
Source: BMC Genomics. 2015 Feb 21;16(1):111. doi: 10.1186/s12864-015-1329-3 (PMC4349310; doi:10.1186/s12864-015-1329-3)
Supplement: Additional file 8: Figure S3. — Expression level of 17 genes involved in five classes by quantitative real-time reverse transcription-PCR (qRT-PCR) at control condition and six time points after Xanthomonas oryzae pv. oryzae (Xoo) PXO349 infection. Showing the expression level of 17 genes involved in five classes by qRT-PCR under control conditions and six time points after Xoo infection. (A) Brassinosteroid signaling-related genes. (B) Genes involved in hypersensitive cell death due to ion fluxes or reactive oxygen species bursts. (C) R genes. (D) Genes involved in ion transport. (E) Genes encoding pathogenesis-related proteins. Error bars indicate standard deviation. Actin was used as an endogenous control. [file 12864_2015_1329_MOESM8_ESM.pdf]

(A)

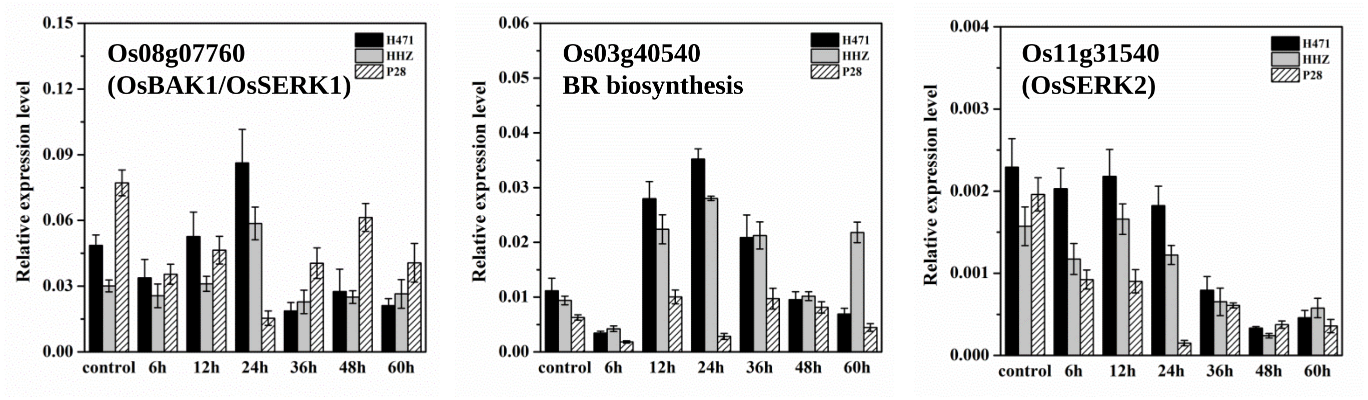
Os08g07760
(OsBAK1/OsSERK1)
Os03g40540
BR biosynthesis
Os11g31540
(OsSERK2)
H471
HHZ
P28
Relative expression level
control
6h
12h
24h
36h
48h
60h

(B)

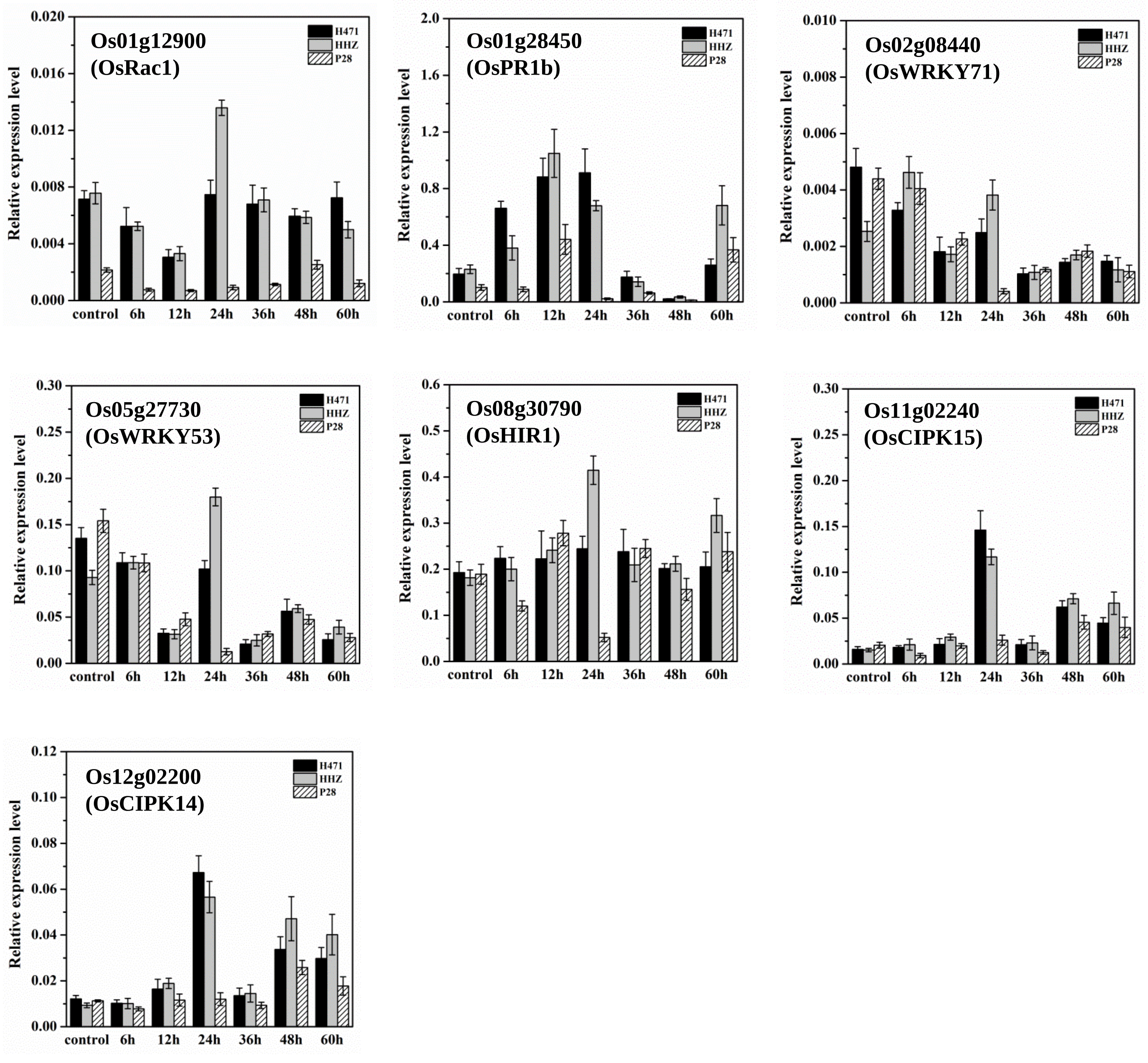
Os01g12900
(OsRac1)
Os01g28450
(OsPR1b)
Os02g08440
(OsWRKY71)
Os05g27730
(OsWRKY53)
Os08g30790
(OsHIR1)
Os11g02240
(OsCIPK15)
Os12g02200
(OsCIPK14)
H471
HHZ
P28
Relative expression level
control
6h
12h
24h
36h
48h
60h

(C)

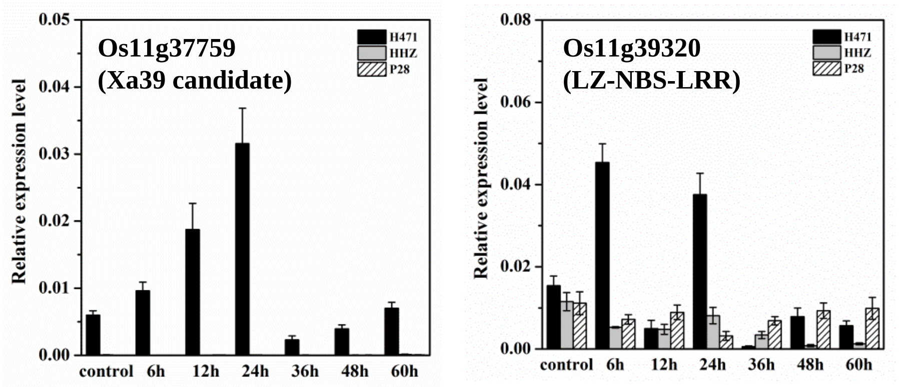

(D)

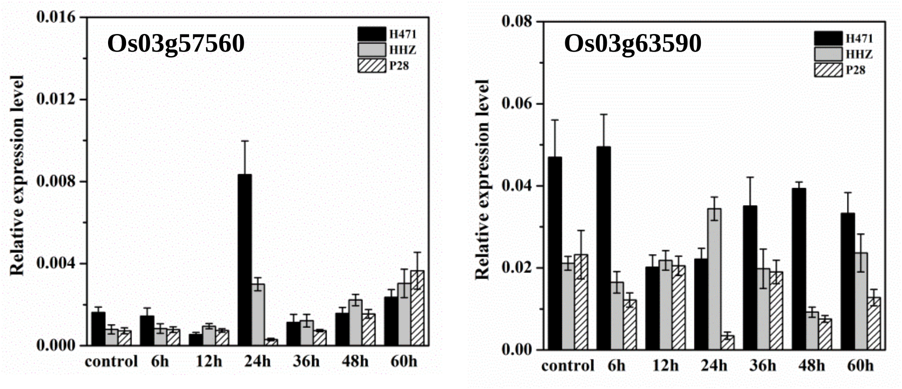

(E)

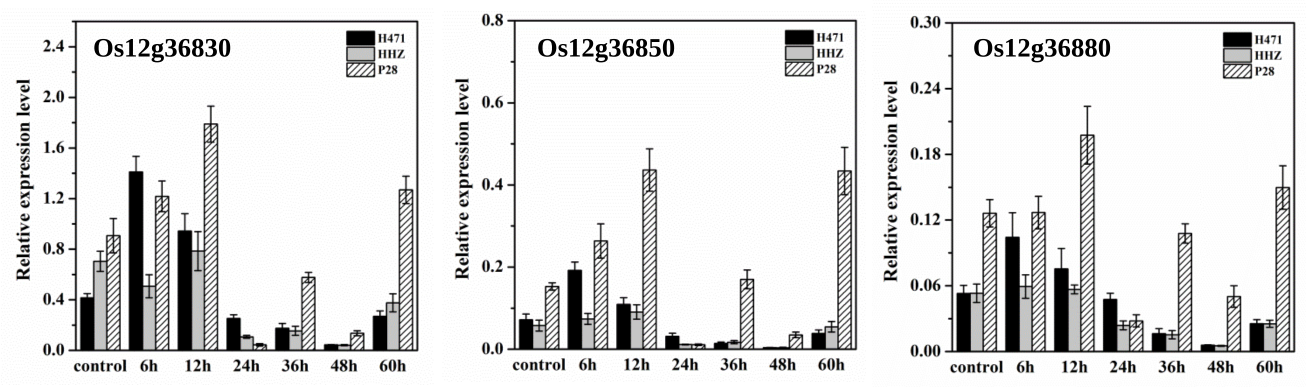

Additional file 8. Expression level of 17 genes involved in five classes by quantitative real-time reverse transcription-PCR (qRT-PCR) at control condition and six time points after *Xanthomonas oryzae* pv. *oryzae* (*Xoo*) PXO349 infection. (A) Brassinosteroid signaling-related genes. (B) Genes involved in hypersensitive cell death due to ion fluxes or reactive oxygen species bursts. (C) *R* genes. (D) Genes involved in ion transport. (E) Genes encoding pathogenesis-related proteins. Error bars indicate standard deviation. Actin was used as an endogenous control.
