# Supplementary material for: Comparative transcriptome profiling of a rice line carrying Xa39 and its parents triggered by Xanthomonas oryzae pv. oryzae provides novel insights into the broad-spectrum hypersensitive response
Source: BMC Genomics. 2015 Feb 21;16(1):111. doi: 10.1186/s12864-015-1329-3 (PMC4349310; doi:10.1186/s12864-015-1329-3)
Supplement: Additional file 10: Table S7. — Differentially expressed defense-related genes in the rice introgression line H471 compared with the recurrent parent Huang-Hua-Zhan (HHZ) infected by Xanthomonas oryzae pv. oryzae PXO349. Containing differentially expressed defense-related genes in H471 compared with HHZ infected by Xanthomonas oryzae pv. oryzae. [file 12864_2015_1329_MOESM10_ESM.docx]

**Addition file 10.** Differentially expressed defense-related genes in the rice introgression line H471 compared with the recurrent parent Huang-Hua-Zhan (HHZ) infected by *Xanthomonas oryzae* pv. *oryzae* PXO349

| **Gene ID** | **Allele source in H471** | **H471-1d vs HHZ-1d** | | **H471-2d vs HHZ-2d** | | **H471-1d vs H471-ck** | | **H471-2d vs H471-ck** | | **MSU Annotation** |
| --- | --- | --- | --- | --- | --- | --- | --- | --- | --- | --- |
|  |  | **log2(FC)^a^** | **Up/Down^b^** | **log2(FC)** | **Up/Down** | **log2(FC)** | **Up/Down** | **log2(FC)** | **Up/Down** |  |
| **Up-regulation** |  |  |  |  |  |  |  |  |  |  |
| LOC_Os03g40194 | HHZ | 1.17 | up | 1.16 | up | 0.21 | -- | -0.03 | -- | NB-ARC domain-containing disease resistance protein |
| LOC_Os04g30930 | heterozygo | 1.29 | up | 0.61 | -- | 0.93 | up | 0.92 | up | NB-ARC domain-containing disease resistance protein |
| LOC_Os08g14810 | heterozygo | inf | up | inf | -- | 1.46 | -- | 0.18 | -- | NB-ARC domain-containing disease resistance protein |
| LOC_Os11g39320 | heterozygo | inf | up | inf | up | 1.24 | up | 0.67 | -- | NB-ARC domain-containing disease resistance protein |
| LOC_Os12g36880 | HHZ | 1.18 | up | 1.11 | up | -0.47 | -- | -2.02 | down | Pathogenesis-related Bet v I family protein |
| LOC_Os12g36830 | HHZ | 1.02 | up | 0.25 | -- | -1.50 | down | -2.76 | down | Pathogenesis-related Bet v I family protein |
| LOC_Os12g36850 | HHZ | 1.65 | up | 1.67 | -- | -2.18 | down | -4.03 | down | Pathogenesis-related Bet v I family protein |
| LOC_Os05g33520 | HHZ | 1.14 | up | 1.21 | up | 0.91 | up | 0.56 | -- | Resistance to phytophthora 1 (NBS-LRR class) |
| LOC_Os11g35850 | HHZ | 2.54 | up | 2.84 | up | -0.11 | -- | -0.25 | -- | Disease resistance family protein / LRR family protein |
| LOC_Os11g37759 | heterozygo | inf | up | inf | up | 1.24 | up | 0.51 | -- | Disease resistance protein (CC-NBS-LRR class) family |
| **Down-regulation** |  |  |  |  |  |  |  |  |  |  |
| LOC_Os03g48320 | HHZ | -4.68 | down | -1.62 | down | -3.94 | down | -0.83 | -- | Disease resistance RPP13-like protein 1 (NB-ARC) |
| LOC_Os06g15730 | HHZ | -0.79 | -- | -1.85 | down | 4.09 | -- | 3.73 | -- | NB-ARC domain-containing disease resistance protein |
| LOC_Os07g03730 | HHZ | -1.38 | down | -0.38 | -- | 2.32 | up | 1.21 | -- | CAP (Cysteine-rich secretory proteins, Antigen 5, and Pathogenesis-related 1 protein) superfamily protein |
| LOC_Os08g07774 | heterozygo | -8.13 | -- | -1.06 | down | -7.51 | -- | -0.22 | -- | Disease resistance protein RPM1 |
| LOC_Os08g07890 | heterozygo | -8.39 | -- | -1.74 | down | -9.04 | -- | -2.20 | down | NB-ARC domain containing protein |
| LOC_Os11g34880 | heterozygo | NA | down | -1.19 | down | NA | down | -0.19 | -- | NB-ARC domain containing protein |
| LOC_Os11g34920 | heterozygo | -8.08 | -- | -1.08 | down | -7.16 | -- | 0.31 | -- | Stripe rust resistance protein Yr10 (NB-ARC) |
| LOC_Os11g34970 | heterozygo | NA | down | -0.58 | -- | NA | -- | -0.08 | -- | Disease resistance protein (CC-NBS-LRR class) family |
| LOC_Os11g37740 | heterozygo | -6.27 | -- | -1.01 | down | -4.90 | -- | 0.18 | -- | Stripe rust resistance protein Yr10 |
| LOC_Os11g38480 | heterozygo | -5.60 | -- | -1.50 | down | -3.50 | -- | 0.91 | -- | NBS-LRR type disease resistance protein |
| LOC_Os11g38520 | heterozygo | NA | down | -1.53 | -- | NA | down | -1.19 | -- | NB-ARC domain-containing disease resistance protein |
| LOC_Os12g38170 | HHZ | -0.95 | down | -0.20 | -- | -4.42 | down | -4.03 | down | Pathogenesis-related thaumatin superfamily protein |

a: The fold change of gene expression is represented by a log2 ratio. b: ‘up’ and ‘down’ means significant differential up-regulated and down-regulated expression ( p-value < 0.001), respectively, ‘inf ’ represents infinite, and ‘NA’ represents not applicable.
