# Supplementary figures and images for: Comparative transcriptome profiling of a rice line carrying Xa39 and its parents triggered by Xanthomonas oryzae pv. oryzae provides novel insights into the broad-spectrum hypersensitive response
Source: BMC Genomics. 2015 Feb 21;16(1):111. doi: 10.1186/s12864-015-1329-3 (PMC4349310; doi:10.1186/s12864-015-1329-3)

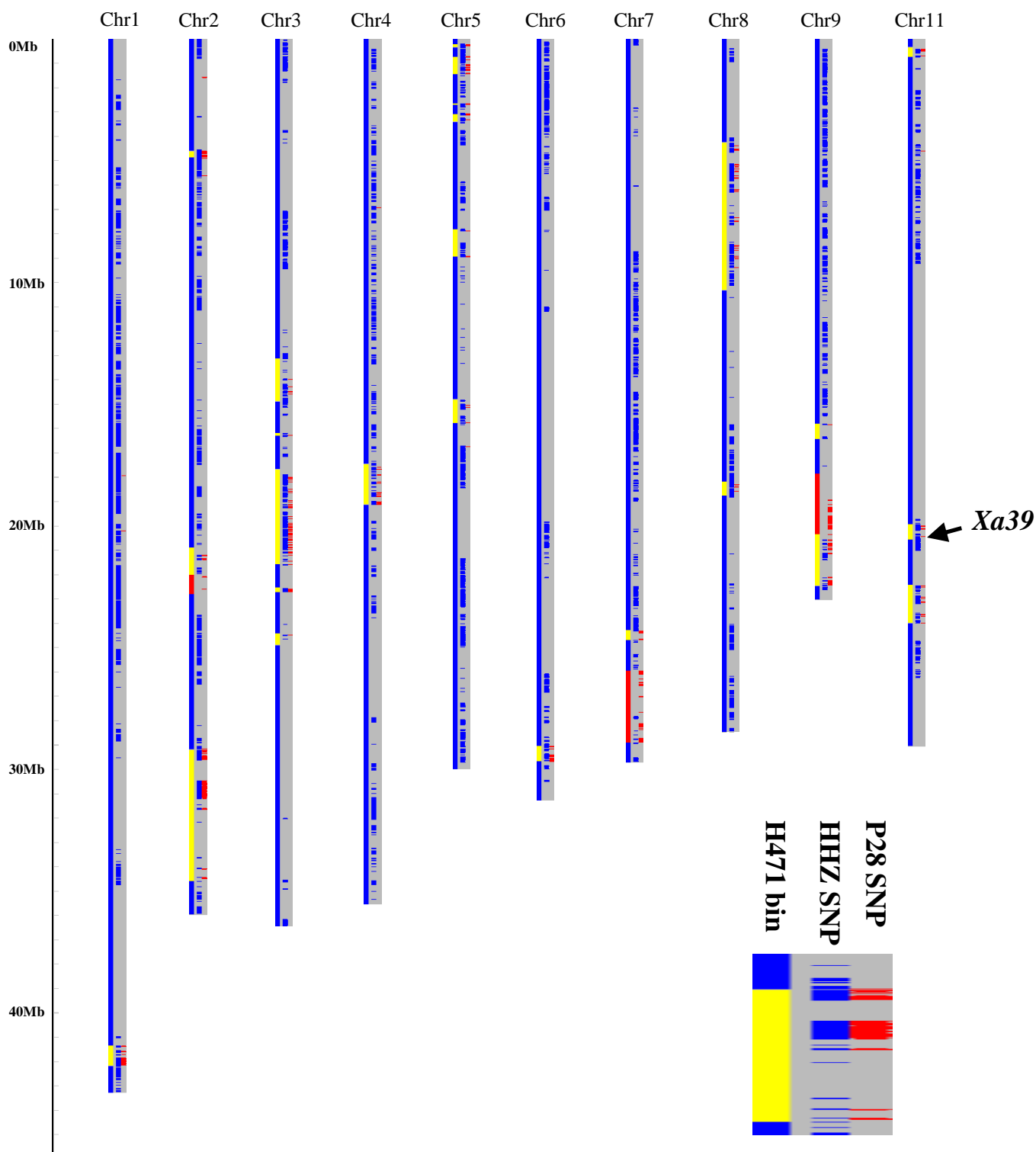

**Additional file 13.** Recombination map of the rice introgression line H471.

Supplement: Additional file 13: Figure S4. — Recombination map of the rice introgression line H471. Containing introgressed bins of H471 based on a genome-wide single nucleotide polymorphism analysis by re-sequencing. [file 12864_2015_1329_MOESM13_ESM.pdf]
